# Biomechanical Analysis of Grafted and Nongrafted Maxillary Sinus Augmentation in the Atrophic Posterior Maxilla with Three-Dimensional Finite Element Method

**DOI:** 10.1155/2020/8419319

**Published:** 2020-10-02

**Authors:** Xuan Wang, Tianqi Zhang, Enli Yang, Zhiyuan Gong, Hongzhou Shen, Haiwei Wu, Dongsheng Zhang

**Affiliations:** ^1^Department of Oral and Maxillofacial Surgery, Shandong Provincial Hospital Affiliated to Shandong First Medical University, Jinan, China; ^2^Department of Oral and Maxillofacial Surgery, Shandong Provincial Hospital Affiliated to Shandong University, Jinan, China

## Abstract

This study is aimed at determining the optimal sinus augmentation approach considering the poor bone condition in the zone of atrophic posterior maxilla. A series of simplified maxillary segment models varying in residual bone height (RBH) and bone quality were established. A 10 mm standard implant combined with two types of maxillary sinus augmentation methods was applied with the RBH, which was less than 10 mm in the maxilla. The maximal equivalent von Mises (EQV) stress in residual bone was evaluated. Bone quality had an enormous impact on the stress magnitude of supporting bone. Applying sinus augmentation combined with grafts was suitable for stress distribution, and high-stiffness graft performed better than low-stiffness one. For 7 mm and 5 mm atrophic maxilla, nongrafted maxillary sinus augmentation was feasible in D3 bone. Poor bone quality was a negative factor for the implant in the region of atrophic posterior maxilla, which could be improved by grafts. Meanwhile, the choice of maxillary sinus augmentation approaches should be determined by the RBH and quality.

## 1. Introduction

Implant restoration is an effective means to restore the configuration and function of missing teeth. Previous studies have demonstrated a high success rate for this kind of therapy, confirming the merits of dental implant treatment [1]. As a load-bearing device, the dental implant needs to sustain the occlusal force and transfer load to the supporting bone, as was determined by the volume of residual bone [2, 3]. In clinical practice, edentulous patients with progressive alveolar bone resorption or adjacent crucial anatomical structure, such as maxillary sinus, make the provision of the implant with optimal dimensions an arduous task [4].

To address this, maxillary sinus augmentation is a predictable surgical method that increases the volume of the available bone [5, 6] and was developed to optimize alveolar bone configuration in the region of the posterior maxilla. Generally, maxillary sinus augmentation can be classified into two types: one is in combination with utilizing graft in maxillary sinus augmentation and the other one is merely treated with maxillary sinus augmentation without using any grafts. Sinus floor augmentation with subsequent graft materials embedding has been attested to be an effective technique for correcting bone deficits by a series of clinical evidence [7]. A wide variety of graft materials are available for restoring the resorbed bone, including autologous bone, alloplasts, xenografts, allografts, or a combination of these [8]. The ideal one could improve bone biomechanical effects to a significant extent. However, the necessity of placing the graft in augmented sinus has been questioned recently [9–11]. Palma et al. [12] declared that with the newly created space following sinus floor augmentation maintaining, the blood clot inside could gain bone formation. A recent systematic review declared that there were no significant differences in the short-time success rate of implant placement between grafted and non-grafted sinus augmentation [13]. Though sinus augmentation has been a commonly applied technique, the choice of sinus augmentation approaches when encountering the atrophic maxilla possessing poor bone quality needs to be further validated.

Available residual bone was particularly crucial in implant placement, described in terms of bone quality and quantity to reflect the bone biomechanical property. Bone quality was related to bone tissue elasticity and mechanical strength, which subsequently determined implant selection and surgical means [14]. The supporting bone possessing high density could not only provide better mechanical stabilization but also permit even stress distribution in the bone-implant interface [15]. However, weak bone quantity or quality was commonly encountered in the region of posterior maxilla, which led to a higher occurrence of failure compared with other regions. In particular, the lower density of trabecular bone can easily reduce the stability of the implant and increase bone stress [15, 16].

At present, the three-dimensional finite element (3-D FE) analysis has been widely used to study the mechanical behavior of implant-related structures and surrounding bone tissue. With the recent advances in this technology, it is possible to simulate complex structures on a microscopic scale to observe further stress distribution that is clinically impossible to observe, to analyze the relevant stresses in the internal structure of the model, and to assess the associated risks [17–19]. The problem of choosing sinus augmentation approaches based on actual bone quality and residual bone volume is of great importance in dental implantation. The 3-D FE analysis was adopted in this study to verify the ultimate magnitude of bone stress in an intricate system of bone tissue. The biomechanical effects of different sinus augmentation methods are examined in a maxilla possessing various strength characteristics. The study is aimed at determining the optimal sinus augmentation method based on the condition of the supporting bone.

## 2. Materials and Methods

### 2.1. FE Models

**Figure 1:**
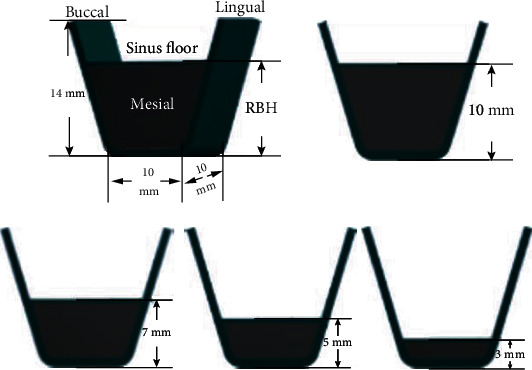
Simplified maxillary segment model. The buccolingual and mesiodistal distance was 10 mm; the overall height was 14 mm. The maxillary models were classified into 4 groups by RBH: 3 mm, 5 mm, 7 mm, and 10 mm.

**Figure 2:**
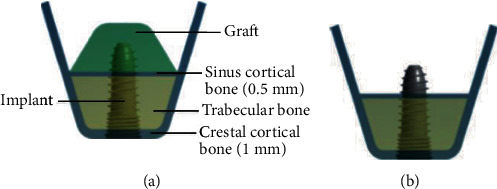
Two types of maxillary sinus augmentation approaches. (a) Grafted sinus augmentation: the apical portion of the implant was totally embedded into the graft; (b) Nongrafted sinus augmentation.

Model a series of simplified maxillary segment models with Creo 2.0 (PTC software, USA). Attempting to simulate the atrophic posterior maxilla, the maxillary models were classified into two types of bone quality, D3 and D4 bone according to Lekholm et al. [20]. Despite the difference in the density of trabecular bone, they both possessed the same configuration and structure. The simplified maxillary segment consisted of thin cortical bone surrounding trabecular bone. The thickness of cortical bone was 0.5 mm in the sinus site and 1 mm in the crestal site. These models have different RBHs. The overall dimension of this segment was 10 mm in mesiodistal length and buccolingual width and 14 mm in height. All maxillary segment models were divided into 4 groups based on the RBH: 3 mm, 5 mm, 7 mm, and 10 mm (Figure 1). For groups with less than 10 mm RBH, the biomechanical effects of grafted and nongrafted maxillary sinus augmentation were investigated. Graft in maxillary sinus was presumed fully peri-implant packing for simulating an ideal situation suggested by Tepper et al. [21]. A titanium implant model (Neo CMI implant, IS 410, Neo Biotech, Korea) was the same as the original mechanical drawing measured with a vernier caliper. The major diameter of the standard implant was 4.0 mm; the total length was 10 mm (Figure 2).

**Figure 3:**
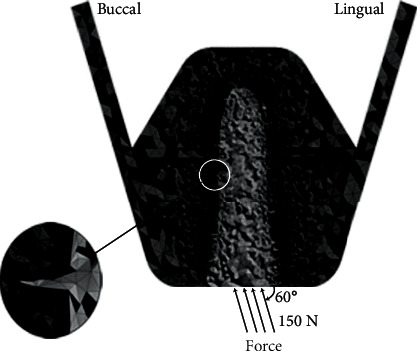
Cross-sectional views of the symmetry plane in the meshed model. A 150 N oblique force was applied to the implant at an angle of approximately 60° to the occlusal surface of the implant.

All 3D solid models were input into a FE software (Ansys Workbench 14.5, SAS IP, Canonsburg, PA) designed to generate and analyze discrete FE meshes. All finite element models were generated from means of tetrahedral elements, and refinement was used to the bone-implant interface of interest to ensure the accuracy of results (Figure 3). Stress distribution in supporting bone was assessed by the maximal EQV stress, which was reported to be a credible parameter for evaluating bone failure risk [16, 22].

### 2.2. Material Properties

In this study, materials were linearly elastic and isotropic, and all materials' volume is considered to be homogenous. Implants were supposed to be made of titanium alloy Ti6Al4V.  Table 1 lists the corresponding modulus of elasticity and Poisson's ratio for all materials [19, 23, 24]. The high-dense trabecular bone's (D3 bone) elastic modulus was 1.37 GPa, and low-dense trabecular bone (D4 bone) was 0.231 GPa. Two distinct grafts were assigned to represent a wide spectrum of stiffness. The higher one is closer to the cortical bone, and the lower one is lower than the stiffness of the high stiffness trabecular bone.

### 2.3. Boundary Conditions and Loading

The exterior nodes on the maxillary segment's mesial and distal surfaces were constrained to no displacement as boundary conditions for each model. A fixed bond was prescribed between the rough implant surface and surrounding bone for simulating the biomechanical environment. A 150 N oblique force was applied to the implant at an angle of approximately 60°to the implant's occlusal surface, as shown in Figure 3[24–26]. The loading force was static and was calculated by EQV stress.

## 3. Results

**Figure 4:**
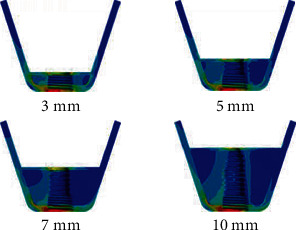
Stress distribution contour map in D3 bone adopting nongrafted sinus augmentation.

**Figure 5:**
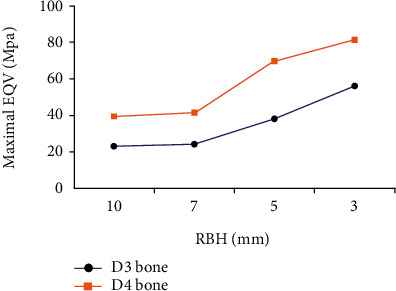
The magnitude of maximal EQV stress in D3 and D4 bone with nongrafted sinus augmentation.

The stress distribution contour map showed that stress concentration was mainly located in the cortical bone around the implant's cervical portion, irrespective of bone condition (Figure 4). Comparative analysis of D3 and D4 bone stresses shows that bone quality has a huge impact on the stress distribution of supporting bone (Figure 5). It was clear that with the reduction of RBH, stress magnitude in D4 bone was markedly higher than D3 bone. The gap tended to enlarge following the aggravation of residual bone resorption.

**Figure 6:**
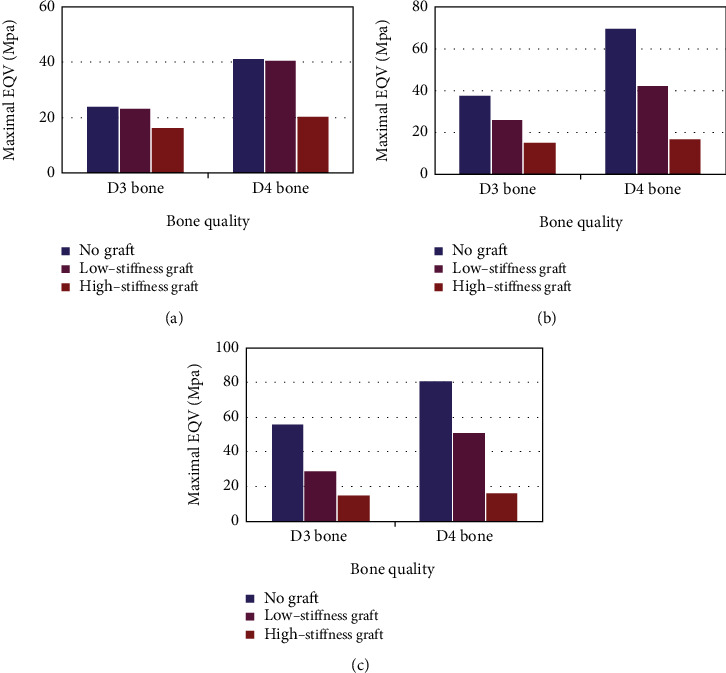
The maximal EQV stress induced by grafted and nongrafted sinus augmentation approaches: (a) 7 mm RBH; (b) 5 mm RBH; (c) 3 mm RBH.

When RBH was 10 mm, residual bone volume was just suitable for the standard implant. The maximal EQV stress was 22.94 MPa in D3 bone and 39.32 MPa in D4 bone. The maximum EQV stress values of the remaining groups were shown in Table 2. When RBH was resorbed to 7 mm, there was only a slight increase in stress values. Applying a low-stiffness graft under this circumstance seemed to do no apparent help for improving stress distribution. On the contrary, the high-stiffness group decreased stress value by nearly 31.6% in D3 bone and 52.2% in D4 bone (Figure 6(a)). When RBH reduced to 5 mm, a rapidly increasing stage of EQV stress emerged. Taking bone quality into account, the stress level in D3 bone with 5 mm RBH was close to that in D4 bone with 10 mm RBH. Nevertheless, the stress level of D3 bone with 3 mm RBH is lower than that of D4 bone with 5 mm RBH. Stress value dropped apparently with the help of low-stiffness graft. For the high-stiffness graft, the decreasing degree of stress value enlarged (Figure 6(b)). When RBH remained only 3 mm, the residual bone was incapable of bearing load solely. The results confirmed this that stress value rose by 143.8% in D3 bone and 106.9% in D4 bone compared with 10 mm group, which was unsatisfactory and made employing graft indispensable. Herewith quite limited residual bone, even the low-stiffness graft could decrease stress value markedly and high-stiffness one performed better (Figure 6(c)). Interestingly, despite the RBH, high-stiffness graft always reduced stress magnitude to the optimal level lower than 10 mm group.

## 4. Discussion

Two kinds of complications occur to the dental implant, namely, mechanical and biological complications, related to adverse biomechanical effects [24]. The excessive load was considered one of the main adverse biomechanical effects contributing to damaging normal bone remodeling equilibrium [27]. Therefore, from the biomechanical point of view, efforts should be made to avoid the adverse biomechanical effects, especially for the atrophic posterior maxillary region. However, the biomechanical effects caused by loading conditions or surrounding bone conditions are challenging to explore by solely applying clinical approaches. With these in mind, FE analysis has been generally used as a complementary approach to study the biomechanical response in bone tissue. This study focused on the biomechanical effects caused by RBH and bone quality.

Notably, despite the changing variables, we observed that the highest overall stress was always concentrated in the implant neck's cortical bone. The same biomechanical behavior was also validated by a couple of prior studies [4, 28, 29]. Koca et al. suggested that the rigid connection between implant and bone accounted for this [2]. Furthermore, such a stress distribution pattern was one of the vital factors involving time-dependent marginal bone loss, inevitable progress jeopardizing implant stability after placement [30]. Marginal bone resorption usually begins in the cortical bone and progresses towards the apical direction. Van Steenberge et al. reported that marginal bone loss was as high as 0.4 mm in the first year after implant implantation, and in the next two years, the annual loss was reduced to 0.03 mm [31]. Hence, it seemed quite essential to diminish stress concentration in supporting bone.

Bone quality is proven to be one of the critical determinants for implant treatment planning [15]. Nevertheless, due to poor bone quality, the implant success rates in the posterior maxillary regions were lower than in other regions [32, 33]. In this case, an attempt was undertaken to investigate the impact of bone quality on the stress magnitude of supporting bone and emphasis on D3 and D4 bone. Although D4 bone possessed the same configuration as for D3 bone, D4 bone was comprised of lower-density trabecular bone inside, which distinguished from D3 bone. Correspondingly, higher stress was found to be located in D4 bone. Kumar demonstrated that the lower-density trabecular bone in D4 bone was incapable of withstanding high stress so that most of the stress was relocated to cortical bone [34]. This was verified by Chang's finding that the unfavorable stress distribution in D4 bone increased the risk of micromotion and instability of implant [24]. Considering the inability of altering the bone quality and consequent rising stress, it is more operable to modify the configuration of the atrophic maxilla to dissipate stress optimally.

Since the introduction of various materials, procedural modifications in the atrophic sinus region had been proposed to be efficient in optimizing stress distribution. As was shown in this study, graft in the maxillary sinus enhanced the load-bearing capacity of residual bone. However, the degree of enhancement mainly depended on its stiffness, which was fairly pertinent to the maturation process to a large extent [30]. The low-stiffness graft exhibited an obvious effect of reducing stress only in 5 mm and 3 mm groups. However, high-stiffness graft diminished stress concentration effectively regardless of different RBH. This was presumably because the loading capacity of the low-stiffness graft was lower than that of high-stiffness one [35]. Therefore, special attention should be paid to the quality of the graft before the implant placement. Having higher stress in surrounding bone with low-stiffness graft embedded into the maxillary sinus would challenge the load-bearing capacity of supporting bone, especially for D4 bone. Additionally, decreasing the graft's stiffness would increase the micromotion in the bone-implant interface during the early stage of implant placement [30]. Hence in the biomechanical aspect, high-stiffness graft might be a better choice for optimizing stress distribution.

Impaired bone height is frequently encountered when placing an implant in the zone of posterior maxilla. The crestal bone atrophy, together with sinus pneumatization in edentulous patients, prevented inserting the implant with an optimal dimension [36]. In order to improve residual bone volume, various surgical techniques and diverse graft materials have been put into use. RBH was the fundamental evaluation criterion determining the choice of sinus augmentation approaches before implant placement [37]. Clinically, when the RBH was above 10 mm, a classic implant procedure was suggested. When the RBH was below 4-5 mm, a surgical approach involving graft materials through a lateral implant placement was recommended [38, 39]. For D3 bone, both two kinds of graft exhibited desirable stress dispersion effects, while stress magnitude in D4 bone was detrimental when applying low-stiffness graft. Consequently, to achieve better quality, sufficient healing duration of the graft was well recommended in the severely atrophic maxilla [35], particularly for D4 bone. Various surgical approaches would be indicated to guarantee implant stability with available RBH ranging from 5 mm to 10 mm. Applying graft materials in sinus augmentation had already achieved affirmative results and become a requisite procedure to remedy alveolar bone deficiency before implant placement [40]. Nevertheless, with the accumulation of successful cases using nongraft sinus augmentation, a systematic review confirmed that the sinus augmentation method without applying any grafts was effective and safe [41]. Judging from the present study, although applying high-stiffness graft could diminish stress, the stress level in the 7 mm group had no significant change when comparing with the 10 mm group. This might mean that the nongrafted sinus augmentation was relatively feasible in the slightly atrophic maxilla. When RBH was reduced to 5 mm, stress appeared to rise markedly. Bruschi et al. [42] asserted that local treatment of the sinus augmentation without utilizing grafts was viable in the region of posterior maxilla, with as little as an RBH of 5 mm. However, according to the present study, stress magnitude was comparatively acceptable only for D3 bone and it turned evidently unfavorable when bone quality deteriorated to D4 bone. For this reason, we speculated that this proposal only applied to maxilla with better bone quality. In general, with the help of grafts, including both low-stiffness and high-stiffness ones, bone stress returned to a tolerable level. Therefore, utilizing grafts to modify bone configuration seemed necessary for moderately atrophic maxilla with poor bone quality.

Because of its unique advantages in stress analysis, the 3D-FE method has been widely used in implant biomechanics research. In this article, we assumed a bond connection in the bone-implant interface. The 3D-FE analysis identified stress differences based on these possible treatment approaches for dental implants in the atrophic posterior maxilla, and the results could still partly demonstrate the biomechanical effects in the intricate biological tissues. However, the results of this study should be extended to the clinical situation. More clinically related models should be designed, and the improved shape of the maxilla will more closely simulate the actual situation for a more reliable FE analysis. Additionally, research is needed to correlate the stress and the response of actual bone tissue with predicted treatment outcomes, and as a way to improve implant design and treatment plans.

## 5. Conclusion

In our finite element study, conclusions can be drawn:

1. For the region of atrophic posterior maxilla, the choice of maxillary sinus augmentation approaches was determined by its residual bone volume and bone quality
2. Graft could optimize stress distribution in supporting bone and its load-bearing capability was largely depended on the stiffness

## Figures and Tables

**Table 1:** Elastic properties of materials modeled.

Material	Young's modulus, *E* (GPa)	Poisson's ration, *ν*
Cortical bone	13.7	0.3
Trabecular bone (D2 & D3)	1.37	0.3
Trabecular bone (D4)	0.231	0.3
High-stiffness graft	11	0.3
Low-stiffness graft	0.5	0.3
Titanium	110	0.35

**Table 2:** Maximal EQV stress (MPa) in supporting bone.

RBH (mm)	No graft		Low-stiffness graft		High-stiffness graft		
	D3	D4	D3	D4	D3	D4
7	24.237	41.268	23.657	40.737	16.567	19.739
5	37.885	69.63	26.139	42.365	15.4	17.031
3	55.927	81.358	29.288	51.583	14.066	16.482

## Data Availability

The data used to support the findings of this study are available from the corresponding author upon request.
